# Protective Impact of Influenza Vaccination on Healthcare Workers

**DOI:** 10.3390/vaccines12111237

**Published:** 2024-10-30

**Authors:** Yimei Tian, Yue Ma, Jianchao Ran, Lifang Yuan, Xianhu Zeng, Lu Tan, Li Chen, Yifan Xu, Shaxi Li, Ting Huang, Hongzhou Lu

**Affiliations:** 1 Department of Preventive Medicine and Healthcare-Associated Infection Management, National Clinical Research Center for Infectious Diseases, Third People’s Hospital of Shenzhen and the Second Affiliated Hospital of Southern University of Science and Technology, No 29 Bulan Road, Longgang District, Shenzhen 518112, China; tym1224@126.com (Y.T.); mayue9999@126.com (Y.M.); ranjianchao@outlook.com (J.R.); zxhlp@126.com (X.Z.); tanlussy2008@163.com (L.T.); 18718687035@163.com (L.C.); xuyifanszsy@163.com (Y.X.); lishaxi1989@163.com (S.L.); 2 School of Nursing, Guangdong Pharmaceutical University, 283 Jianghai Avenue, Guangzhou 510310, China; yuanlifang@gdpu.edu.cn

**Keywords:** influenza, influenza vaccine, healthcare workers, influenza-like illness, vaccine efficacy

## Abstract

Background: Influenza vaccine uptake among healthcare workers is crucial for preventing influenza infections, yet its effectiveness needs further investigation. Objectives: This prospective observational study aimed to assess the protective effect of influenza vaccination among healthcare workers in Shenzhen. Methods: We enrolled 100 participants, with 50 receiving the 2023–2024 quadrivalent influenza vaccine (QIV) and 50 serving as unvaccinated controls. Epidemiological data were collected when the participants presented influenza-like illness. Serum samples were collected at three time points (pre-vaccination and 28 and 180 days after vaccination). Hemagglutination inhibition (HI) assay was performed against the strains included in the 2023–2024 QIV (H1N1, H3N2, BV and BY strains) to assess antibody protection levels. Demographics comparisons revealed no significant differences between the vaccinated and control groups (*p* > 0.05), ensuring group comparability. Results: The incidence of influenza-like illness was significantly lower in the vaccinated (18%) compared to the control group (36%; *p* = 0.046; OR = 0.39; 95% CI: 0.15 to 0.98). The vaccinated group also exhibited a higher rate of consecutive two-year vaccinations (48% vs. 24% in the control group, *p* < 0.05). Additionally, the vaccinated healthcare workers were more inclined to recommend vaccination to their families (80% vs. 48%, *p* < 0.05). HI titers against H1N1 (*p* < 0.01), H3N2 (*p* < 0.01), BV (*p* < 0.001) and BY (*p* < 0.01) significantly increased in the vaccinated group at 28 days post-vaccination. Moreover, a marked and sustained increase in HI titers against the H3N2 strain (*p* < 0.001) was observed at 180 days post-vaccination, highlighting the vaccine’s enduring impact on the immune response. The fold change in the HI titers, indicative of the magnitude of the immune response, was significantly higher for H1N1 (*p* < 0.01), H3N2 (*p* < 0.001), BV (*p* < 0.01) and BY (*p* < 0.05) among the vaccinated individuals compared to the control group, underscoring the vaccine’s efficacy in eliciting a robust and sustained antibody response. Conclusion: Influenza vaccination significantly reduces the incidence of influenza-like illness among healthcare workers and promotes a sustained immune response. The study supports the importance of annual vaccination for this group to enhance personal and public health.

## 1. Introduction

Influenza, as one of the most devastating respiratory infectious diseases, has ignited times of worldwide pandemics for more than a century [1,2]. Continuing concerns about influenza have been brought about by the universal high incidence of influenza [3,4]. Data from the World Health Organization (WHO) revealed around a billion cases of seasonal influenza annually. It causes 290,000 to 650,000 respiratory deaths annually [5,6,7,8]. A population-based study showed that influenza was associated with a substantial excess in respiratory mortality in China [9]. As reported, influenza viruses are an important cause of severe disease, mortality and hospitalizations [10,11]. Compared to the general population, healthcare workers are at a higher risk of influenza infection [12,13]. Influenza infection may lead to increased absenteeism, presenteeism and the disruption of medical services [14,15]. Moreover, the influenza infection of healthcare workers may induce nosocomial infections in patients, which could cause severe complications [16,17].

Influenza vaccination is proven to avert substantial influenza-associated disease including hospitalizations and deaths [18,19,20,21,22]. As a simulation result suggested, when the vaccine effectiveness is 40–60%, the vaccine could control the spread of influenza virus in China if the coverage reaches 40–60% [23]. Healthcare workers are considered a high-risk group for influenza infection due to their occupational exposure to influenza virus [24,25]. The WHO recommends healthcare workers as a priority group for annual influenza vaccination [26,27]. Especially during the COVID-19 pandemic, seasonal influenza vaccination was recommended to health workers to prevent dual infection [28,29]. In order to reduce infection rates and prevent mortality among patients, influenza vaccines are offered to healthcare workers annually in many countries [30,31]. A mandatory influenza vaccine policy has been implemented in America for ten years, which has had a positive impact on the safety of patients [32]. In China, influenza vaccines for healthcare workers have had a moderate effectiveness in preventing influenza [33]. In England, most National Health Service Trusts are implementing interventions as part of their flu program to improve influenza vaccination [34]. However, influenza vaccination coverage among healthcare workers in some counties is not satisfactory [35,36]. The influenza vaccination of healthcare workers has been proven to prevent influenza pandemic, death, health service use and influenza-like illness [37,38]. As indicated, a shielding effect of more than 35% of vaccinated healthcare workers on hospital-acquired influenza among patients in acute-care units was found in a case–control study [39]. The influenza vaccination of healthcare workers is highly suggestive of hospital-acquired flu prevention among hospitalized patients [38,40].

In addition, the sufficient protective effect of vaccines is crucial for driving vaccination initiatives [41]. Safety concerns and insufficient awareness are the main difficulties in the promotion of influenza vaccine among healthcare workers and health-related administrators [42,43,44]. However, the precise immune protection they offer to healthcare workers, who are at the forefront of patient care, is not fully understood. In this study, we established a prospective observational study of influenza vaccination among healthcare workers to illuminate the real-world effectiveness of influenza vaccines. By evaluating the vaccine’s impact on influenza-like illness within this group, we aim to bolster the evidence base that guides vaccination strategies for policymakers and healthcare providers, ensuring the strategic use of vaccines and highlighting their indispensable role in protecting the health of healthcare workers and the broader community they serve.

## 2. Materials and Methods

### 2.1. Study Approval

This study was conducted in a comprehensive third-grade class-A hospital in Shenzhen, China, which includes 2400 beds. This hospital is responsible for the medical treatment and scientific research of infectious diseases. Healthcare workers from departments at a higher risk of influenza infection were recruited in this study to elucidate the preventive effect of vaccines in real working conditions. All the participants were free to opt for vaccination or not. The time period of the recruitment lasted a month (from 20 September to 19 October 2023). Written informed consent was obtained, making sure all the participants were fully informed about our research program and purpose. All the procedures of this research are approved by the Institutional Review Board of the Third People’s Hospital of Shenzhen (No. 2023-073).

### 2.2. Subjects and Study Design

Healthcare workers between 20 and 60 years old were eligible to participate in this prospective observational study. A total of 100 participants were recruited, including 23 men and 77 women. The participants were divided into doctors, nurses and medical technicians. The participants vaccinated with a commercial quadrivalent influenza vaccine (QIV, Shenzhen Sanofi Pasteur Biological Products Co., Ltd., Shenzhen, China) were defined as the vaccinated group (*n* = 50). The participants without the influenza vaccination were categorized as the control group (*n* = 50). All the subjects were asked to complete a questionnaire, which is described in Table 1.

From both the vaccinated and control subjects, fresh blood samples were collected before the vaccination (d0), on day 28 post-vaccination (d28) and on day 180 post-vaccination (d180). Since the control group did not undergo a vaccination, their blood samples were collected at the corresponding time points (d0, d28, d180) to allow for a comparative analysis with the vaccinated group. Then, sera were separated from clotted blood and stored at −80 °C until they were analyzed. A total of 300 serum samples were collected and analyzed in this study.

### 2.3. Follow-Up Observation

The health conditions of all the subjects were followed up weekly for a period of 6 months post-enrollment. Epidemiological data were collected when the participants presented influenza-like illness. The influenza-like illness was defined as a measured fever (≥37.5 °C) and at least one respiratory symptom (cough, sore throat, runny nose, chill, fatigue, headache and dizziness) [45].

### 2.4. Viruses

The strains of 2023–2024 northern hemisphere QIV include A/Victoria/2570/2019 (H1N1), A/Darwin/9/2021 (H3N2), B/Austria/1359417/2021 (B/Victoria, BV) and B/Phuket/3073/2013 (B/Yamagata, BY). All the influenza viruses used in this study were obtained from the Chinese Center for Disease Control and Prevention in Beijing.

### 2.5. Hemagglutination Inhibition (HI) Assay

To assess the HI antibody titer, the serum samples were initially diluted at a ratio of 1:5 in a receptor-destroying enzyme (RDE) from Denka Seiken, Japan, and maintained at 37 °C for 16–18 h. Subsequently, the samples were inactivated at 56 °C for 30 min. The HI assay was conducted using 1% chicken erythrocytes, along with four hemagglutination (HA) units of the viruses (H1N1, H3N2, BV and BY), following the WHO standard procedures [5]. Serial dilutions of the serum were prepared to determine the highest dilution that completely inhibited the hemagglutination, with the reciprocal of this dilution recorded as the HI titer. Each assay was performed in triplicate for reproducibility. The recorded HI titers represent the highest serum dilution that demonstrated the complete inhibition of hemagglutination.

### 2.6. Statistical Analysis

Statistical analysis was performed by using SPSS software 26.0 (SPSS Inc., Chicago, IL, USA). The comparisons of age and BMI between the vaccination and control groups were conducted using the nonparametric Mann–Whitney U test. The comparisons of the categorical variables (demographic characteristics, number of influenza-like illnesses, vaccination history and vaccination recommendations) were conducted by the chi-square test or single-factor logistic regression analysis. The associations between the influenza vaccination and the odds of influenza-like illness were investigated by logistic regression. Adjustments were made for vaccination, age and staff type in the muti-factor logistic regression analysis. A two-sided *p*-value < 0.05 was considered statistically significant. The HI titers were subjected to either a paired or an unpaired *t*-test analysis, depending on the group’s normality and homogeneity of variance. The results were displayed as medians and interquartile ranges (IQR) for the continuous variables and number (percentage) for the categorical variables, with a statistical significance set at a *p*-value < 0.05.

## 3. Results

### 3.1. Characteristics of Participants

Demographic characteristics, including the age, sex, education, professional title, staff type, body mass index (BMI), smoking, alcohol drinking, steps, sleeping status, stress and history of chronic disease of all the subjects, are presented in Table 1.

The medians of the age were 35 years (IQR = 10) and 39 years (IQR = 11), respectively, in the control and vaccination group. In the control group, the highest proportion of age distribution was 31–40 years. In contrast, the age distributions of 20–30 and 31–40 years were the same in the vaccination group. In the sex distribution, the largest proportion was female, both in the control and vaccination groups. In the education levels, the largest proportion was college. In the professional titles, the largest proportion was medium. In the staff types, the highest proportion was doctor in the control group but nurse in the vaccination group. The medians of the BMI were 22.65 (3.45) and 22.13 (3.74), respectively, in the control and vaccination groups. In the BMI categories, the largest proportion was normal both in the control and vaccination groups. The highest proportion of smoking was never in the two groups. The highest proportion of alcohol was also never in the two groups. In the steps levels, the highest proportion was exceeding 10,000 steps in the control group but 5000–10,000 steps in the vaccination group. The highest proportion of sleep duration was 6–8 h per day in the two groups. The highest proportion of stress levels was moderate in the two groups. The highest proportion of the likelihood of chronic disease history was no in the two groups. No significant demographic differences were observed between the vaccination and control groups (*p* > 0.05), indicating the comparability of the two groups across all the assessed variables.

### 3.2. The Incidence of Influenza-like Illness in Both the Control Group and Vaccination Group

Table 2 illustrates the prevalence of influenza-like illness (ILI) among the study participants. In the control group, 36% reported experiencing ILI, while the vaccination group showed a lower proportion of ILI (18%). The incidence of ILI in the control group was on the borderline of significantly higher at 36%, compared to the vaccination group at 18% (*p* = 0.046). In the single-factor logistic regression analysis, the estimated relative odds (odds ratio, “OR”) of ILI in the vaccinated group compared to the control group was 0.39 (95% CI: 0.15 to 0.98), with *p* = 0.046 for the null hypothesis that OR = 1. Statistically significantly higher odds of ILI were observed among the subjects aged 20 to 30 years (OR = 4.11; 95% CI: 1.08 to15.63) and lower odds were observed among the nurses (OR = 0.20; 95% CI: 0.05 to 0.86).

### 3.3. The Vaccination History and Vaccination Recommendations in Both Control Group and Vaccinated Group

Data on vaccination history and recommendations were gathered through a questionnaire survey, as detailed in Table 3. The control group had a 40% rate of never having been vaccinated, compared to 24% in the vaccination group. For one-year vaccination rates, the control group reported 36%, while the vaccination group reported 28%. Notably, the vaccination group had a significantly higher rate of vaccination for two consecutive years (48%) than the control group (24%, *p* < 0.05).

Table 4 highlights that the healthcare workers in the vaccination group were significantly more inclined to recommend vaccinations to their families (80% vs. 48%, *p* < 0.05). Regarding vaccination recommendations, the control group’s responses were distributed as follows: strongly recommend (56%), with reservations (40%), and not recommend (4%). In contrast, the vaccination group showed a higher willingness to strongly recommend vaccination: 78% strongly recommended it, 20% had reservations, and 2% did not recommend it. For family vaccination recommendations, the control group had 48% strongly recommend, 48% with reservations, and 4% not recommend, while the vaccination group was more decisive: 80% strongly recommended it, 20% had reservations, and none did not recommend it.

### 3.4. Muti-Factor Logistic Regression Analysis on Incidence of Influenza-like Illness in Healthcare Workers

In a multi-factor logistic regression model with vaccination, age and staff type as the independent variables, none of these were significantly associated with the dependent variable ILI (*p* > 0.05 for all three null hypotheses that OR = 1). Age had the highest *p*-value (*p* = 0.141), so we removed it from the model. The multi-factor logistic regression analysis presented in Table 5 examines the odds of ILI as the dependent variable, with vaccination status and staff type as independent variables. Our findings indicate that vaccination acts as a protective factor against ILI among healthcare workers. The OR for vaccination is 0.39, which suggests that vaccinated healthcare workers have approximately 61% lower odds of experiencing ILI compared to their unvaccinated counterparts. However, the 95% confidence interval (CI) for the OR extends from 0.15 to 1.00, encompassing the null value of 1. This indicates a protective effect of vaccination but with only a borderline statistical significance. Regarding staff type, the OR is 1.10, with a 95% CI ranging from 0.54 to 2.23. This range suggests that there is no significant impact of staff type on the odds of ILI independent of vaccination status.

### 3.5. Robust Antibody Response Were Observed After QIV Vaccination

**Figure 1:**
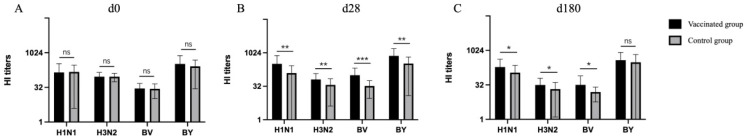
Comparison of the hemagglutination inhibition (HI) titers against the 2023–2024 quadrivalent influenza vaccine (QIV) strains on different time points between the vaccinated and control groups. (**A**) The HI titers in response to H1N1, H3N2, BV, and BY strains were detected on the day before vaccination (d0) in the vaccinated and control groups. (**B**) The HI titers in response to H1N1, H3N2, BV, and BY strains were detected on day 28 (d28) post-vaccination in the vaccinated and control groups. (**C**) The HI titers in response to H1N1, H3N2, BV, and BY strains were detected on day 180 (d180) post-vaccination in the vaccinated and control groups. Data are presented as the mean ± deviation (SD) and analyzed using an unpaired-sample *t*-test. *p* < 0.05 was considered significantly different. * *p* < 0.05; ** *p* < 0.01; *** *p* < 0.001; ns, no significance.

Serum samples were collected at three distinct time points: the day prior to the vaccination (d0), 28 days post-vaccination (d28) and 180 days post-vaccination (d180). At d0, the initial HI titers for H1N1, H3N2, BV and BY showed no significant differences between the vaccinated and control groups (Figure 1A). At d28, the vaccinated group exhibited a significant increase in the HI titers for H1N1 (*p* < 0.01), H3N2 (*p* < 0.01), BV (*p* < 0.001) and BY (*p* < 0.01) compared to the control group, indicating a robust antibody response following the QIV vaccination (Figure 1B). By d180, the HI titers for H1N1 (*p* < 0.05), H3N2 (*p* < 0.05) and BV (*p* < 0.05) remained significantly increased in the vaccinated group compared to the control group. However, the HI titer for BY had no significant difference between these two groups (Figure 1C). Despite this, a strong antibody response was still evident at d180 after the QIV vaccination.

The HI titers in response the to H1N1, H3N2, BV and BY strains were detected on the day before the vaccination (d0) (A) and at day 28 (d28) (B) and day 180 (d28) (C) post-vaccination in the vaccinated and control groups. The data are presented as the mean ± deviation (SD) and analyzed using an unpaired-sample *t*-test. *p* < 0.05 was considered significantly different. * *p* < 0.05; ** *p* < 0.01; *** *p* < 0.001; and ns, no significance.

**Figure 2:**
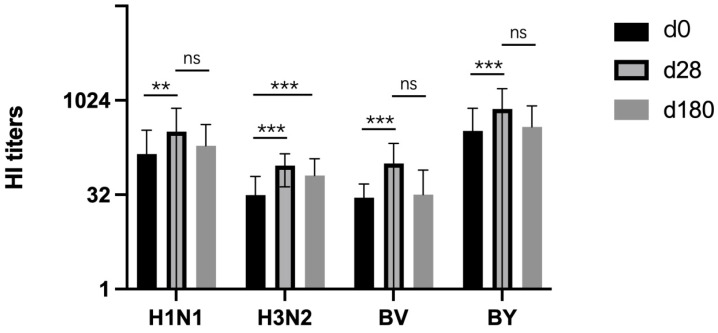
Progression of the hemagglutination inhibition (HI) titers over time in the vaccinated group following the 2023–2024 quadrivalent influenza vaccine (QIV) vaccination. ** *p* < 0.01; *** *p* < 0.001; ns, no significance.

Moreover, we assessed the variation in the HI antibody titers within the vaccination group at different time points post-vaccination. For H1N1, there was a significant increase in the HI titer at d28 (*p* < 0.01), but no significant difference was observed at d180 compared to d0 (Figure 2). For H3N2, significant increases in the HI titer were observed at both d28 (*p* < 0.001) and d180 (*p* < 0.001) when compared to d0 (Figure 2). Similarly, for both BV and BY, significant increases in the HI titer were noted at d28 (*p* < 0.001 for both) in comparison to d0 (Figure 2). However, no significant difference was observed at d180 compared to d0. The above results indicate that the QIV vaccination can increase the antibody titers of all the subtypes in the serum.

The HI titers in response to the H1N1, H3N2, BV and BY strains were detected on the day before the vaccination (d0) and at day 28 (d28) and day 180 (d180) post-vaccination in the vaccinated group. The data are presented as the mean ± deviation (SD) and analyzed using a paired-sample *t*-test. *p* < 0.05 was considered significantly different. ** *p* < 0.01; *** *p* < 0.001; ns, no significance.

**Figure 3:**
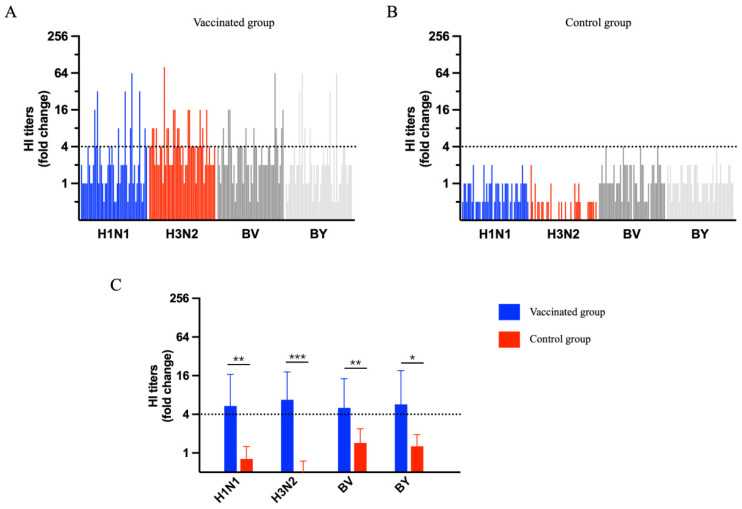
The fold change in the hemagglutination inhibition (HI) titers response to the 2023–2024 quadrivalent influenza vaccine (QIV) strains on day 28 post-vaccination in the vaccinated and control groups. (**A**) Individual fold changes in HI titers in response to H1N1, H3N2, BV, and BY strains on day 28 post-vaccination in the vaccinated group. (**B**) Individual fold changes in HI titers in response to H1N1, H3N2, BV, and BY strains on day 28 post-vaccination in the control group. (**C**) Comparison of the fold change in HI titers response to H1N1, H3N2, BV, and BY strains on day 28 between the vaccinated and control groups. Data are presented as the mean ± deviation (SD) and analyzed using a paired-sample *t*-test. P < 0.05 was considered significantly different. * *p* < 0.05; ** *p* < 0.01; *** *p* < 0.001.

Furthermore, we analyzed the fold changes in the HI titers between d28 and d0 to evaluate the protective efficacy of the QIV. In the vaccinated group, most of the fold changes for H1N1, H3N2, BV and BY were more than double by d28 (Figure 3A). Conversely, in the control group, the fold changes for these subtypes were less than double by d28 (Figure 3B). Compared to the control group, the vaccinated group showed a significantly greater multiple-fold increase in the antibody titers for H1N1 (*p* < 0.01), H3N2 (*p* < 0.001), BV (*p* < 0.01) and BY (*p* < 0.05) on the 28th-day post-vaccination; this suggests that receiving the QIV vaccination provides a more pronounced antibody protection than not being vaccinated.

There were individual fold changes in the HI titers in response to H1N1, H3N2, BV and BY strains on day 28 post-vaccination in the vaccinated (A) and control (B) groups. (C) shows a comparison of the fold change in the HI titers response to the H1N1, H3N2, BV and BY strains on day 28 between the vaccinated and control groups. The data are presented as the mean ± deviation (SD) and analyzed using a paired-sample *t*-test. *p* < 0.05 was considered significantly different. * *p* < 0.05; ** *p* < 0.01; and *** *p* < 0.001.

## 4. Discussion

This study evaluated the protective efficacy of influenza vaccination among healthcare workers in Shenzhen. We observed the incidence of ILI to be twice as high in the control group when compared to the vaccinated group. The disparity is on the borderline of being statistically significant, but it is large enough to be of practical importance to healthcare workers. With 50 subjects in each group and the threshold of statistical significance set at *p* = 0.05 (two-sided, alpha = 0.05), we had a 53% power (beta = 0.47) to detect a two-fold disparity of incidence between the two groups. We likely could have unequivocally demonstrated statistical significance if we had recruited larger groups of study subjects. With 94 subjects in each group, the power would have been 80%. Also, we did not serologically confirm the reported cases of influenza-like illnesses among the study subjects, and the definition of ILI would include cases of non-influenza respiratory illnesses, against which the QIV could not have been expected to have any protective effect. The occurrence of non-influenza illness among both the vaccinated and control groups likely masked a larger relative disparity of the incidence of true influenza between the two groups. Notably, the vaccinated group exhibited a higher rate of receiving the vaccine for two consecutive years. Additionally, the healthcare workers from the vaccinated group were more likely to endorse and recommend vaccination to their family members. The HI assay results demonstrated a significant increase in the antibody protection levels against the H1N1, H3N2, BV and BY strains at 28 days post-vaccination in the vaccinated group. Furthermore, an elevated HI titer against the H3N2 strain was observed at 180 days post-vaccination. This prospective clinical study underscores the sustained effectiveness of the influenza vaccine in healthcare workers in real-world settings, reinforcing the significance of annual influenza vaccination for this critical workforce.

To mitigate the risk of nosocomial influenza infections and safeguard public health, the World Health Organization (WHO) advocates for annual influenza vaccinations among healthcare workers. Our study’s findings align with global trends, demonstrating a reduction in influenza-like illness (ILI) incidence post-vaccination, likely due to the high rates of repeated influenza vaccinations [46]. Annual vaccinations may enhance immunogenicity and the preventive effects of influenza vaccines [47]. In this study, we identified a protective effect of prior influenza vaccination among healthcare workers, with the ILI incidence at 36% in the control group and 18% in the vaccinated group, similar to the rates reported in a Saudi Arabian study [48]. However, a higher influenza-positive rate in the general population post-vaccination was noted in a U.S. study [49], suggesting potential variations in the vaccine effectiveness between healthcare workers and the general public. It is also possible that asymptomatic infections are under-reported, which could skew the observed ILI rates.

In addition, influenza vaccination among healthcare workers would increase the confidence of the general public with a typical and exemplary role [50,51]. In this study, the vaccinated healthcare workers were more likely to recommend vaccination to their families, potentially increasing vaccine acceptance and uptake in the general population. Strategies to boost influenza vaccination rates among healthcare workers are gaining international attention. In the U.S., mandatory vaccination policies have been instrumental in increasing vaccination rates [52]. Since 2018, the Chinese Center for Disease Control and Prevention has issued technical guidelines for influenza vaccination in China [53,54,55]. In 2023, healthcare workers were added to the priority groups [56]. In Hong Kong, personal coaching was found to increase the vaccination rates among healthcare workers before and during the COVID-19 pandemic [57].

The effectiveness of vaccines is intrinsically linked to the induction of antibodies, which is a critical component of protection against infectious diseases. Influenza, in particular, is primarily thwarted by the host’s antibody response, as evidenced by the significant increase in hemagglutination inhibition (HI) titers post-vaccination, which is indicative of a robust humoral immune response [58,59]. In our study, we observed a marked elevation in the HI titers for the QIV strains at d28 post-vaccination, underscoring the swift antibody response elicited by the vaccine. This finding is consistent with the rapid induction of antibodies following immunization, which is pivotal for immediate protection against influenza strains. Additionally, the sustained increase in the HI titers for the H1N1, H3N2 and B/Victoria (BV) strains at day 180 post-vaccination suggests a long-lasting antibody production, potentially offering protection for an extended period (~180 days). This prolonged immune response is particularly noteworthy, as it implies that the vaccine may provide durable immunity, which is a desirable attribute for seasonal influenza vaccines. However, the HI titer levels for the BY strain did not exhibit significant differences between the vaccinated and control groups at d180. This observation warrants further investigation, as it may be attributed to several factors, including the antigenic properties of the BY strain or variations in the immune response among individuals. It is also plausible that the BY strain may require a different or additional immune response strategy for effective protection. Our study also considered the role of preexisting high serum antibody levels, which have been correlated with the response to influenza vaccination [60]. The initial HI titers for H1N1, H3N2, BV and BY showed no significant differences between the vaccinated and control groups, suggesting that the observed antibody response was likely due to the vaccine rather than pre-existing immunity. This finding is crucial as it implies that the vaccine’s efficacy is not confounded by the baseline antibody levels of the recipients. The variation in the HI antibody titers within the vaccinated group at different time points post-vaccination highlights the dynamic nature of the immune response and the need for a comprehensive understanding of the factors influencing it. This variability could be influenced by numerous factors, including age, underlying health conditions and genetic predispositions, which are all important considerations for vaccine development and administration strategies. While the HI assay is a valuable tool for assessing humoral immunity, it is also essential to consider other aspects of the immune response, such as cell-mediated immunity, which may play a crucial role in long-term protection against influenza. Future studies should aim to evaluate the interplay between antibody and cellular responses to provide a more holistic understanding of vaccine-induced immunity.

However, there are several limitations in this study. The healthcare workers in this study were just from one hospital, which lacked representation. Further research with large populations from different cities are needed to verify our findings. In addition, the influenza-like illness cases in this study were not confirmed and could be similar diseases such as COVID-19. In future studies, we would constantly improve the research design [61].

## 5. Conclusions

Our study provides valuable insights into the immune response following QIV vaccination, highlighting the importance of a robust and sustained antibody response for effective protection against influenza. The incidence of influenza-like illness was significantly decreased after the vaccination. In addition, the vaccination group were more inclined to recommend vaccination to their families. However, the observed differences in the response to the BY strain and the need for a broader assessment of immune responses underscore the complexity of vaccine-induced immunity and the necessity for continued research in this area.

## Figures and Tables

**Table 1:** Subject demographics of the study population [n (%) or medians (IQR)].

Characteristics	Total (*n* = 100)	Control (*n* = 50)	Vaccination (*n* = 50)	*p*
Age (years)	36 (10)	35 (10)	39 (11)	0.02 *
				0.51 **
20–30	24 (24.00)	14 (28.00)	10 (20.00)	
31–40	49 (49.00)	29 (58.00)	20 (40.00)	
≥41	27 (27.00)	7 (14.00)	20 (40.00)	
Sex				0.48 **
Male	23 (23.00)	13 (26.00)	10 (20.00)	
Female	77 (77.00)	37 (74.00)	40 (80.00)	
Education				0.08 **
<College	12 (12.00)	3 (6.00)	9 (18.00)	
College	54 (56.00)	26 (52.00)	28 (56.00)	
>College	34 (34.00)	21 (42.00)	13 (26.00)	
Professional title				0.23 **
Primary	17 (17.00)	10 (20.00)	7 (14.00)	
Medium	67 (67.00)	35 (70.00)	32 (64.00)	
Senior	16 (16.00)	5 (10.00)	11 (22.00)	
Staff type				0.26 **
Doctor	42 (42.00)	25 (50.00)	17 (34.00)	
Nurse	48 (48.00)	21 (42.00)	27 (54.00)	
Medical technician	10 (10.00)	4 (8.00)	6 (12.00)	
BMI (kg/m^2^)	22.50 (3.75)	22.65 (3.45)	22.13 (3.74)	0.34 *
				0.55 **
<18.5 (thin)	7 (7.00)	5 (10.00)	2 (4.00)	
18.5 to 24 (normal)	65 (65.00)	31 (62.00)	34 (68.00)	
24 to 28 (obese)	17 (17.00)	8 (16.00)	9 (18.00)	
≥28 (obesity)	11 (11.00)	6 (12.00)	5 (10.00)	
Smoking				0.65 **
Never	95 (95.00)	48 (96.00)	47 (94.00)	
Light	5 (5.00)	2 (4.00)	3 (6.00)	
Alcohol drinking				0.59 **
Never	73 (73.00)	36 (72.00)	37 (74.00)	
Former	4 (4.00)	3 (6.00)	1 (2.00)	
Light	23 (23.00)	11 (22.00)	12 (24.00)	
Steps				0.60 **
<5000 steps	9 (5.00)	4 (8.00)	5 (10.00)	
5000 to 10,000 steps	46 (46.00)	21 (42.00)	25 (50.00)	
≥10,000 steps	45 (45.00)	25 (50.00)	20 (40.00)	
Sleeping status				0.63 **
<6 h/day	2 (2.00)	1 (2.00)	1 (2.00)	
6 to 8 h/day	76 (76.00)	36 (72.00)	40 (80.00)	
≥8 h/day	22 (22.00)	13 (26.00)	9 (18.00)	
Stress				0.91 **
Fewer	6 (6.00)	3 (6.00)	3 (6.00)	
Moderate	60 (60.00)	29 (58.00)	31 (62.00)	
More	34 (34.00)	18 (36.00)	16 (32.00)	
History of chronic disease Family history of chronic disease				0.46 ***
No	92 (92.00)	45 (90.00)	47 (94.00)	
Yes	8 (8.00)	5 (10.00)	3 (6.00)	

Note: * indicates *p*-values derived from the Mann–Whitney rank–sum test. ** indicates *p*-values derived from the chi-square test. *** indicates *p*-values derived from the logistic regression.

**Table 2:** Incidence of influenza-like illness [n (%)] and single-factor logistic regression analysis.

Characteristics	Incidence of Influenza- like Illness [n (%)]	B	SE	Wald	p	OR	95% CI
**Vaccination**							
Yes	9 (18.00)	−0.94	0.47	3.98	0.046	0.39	0.15, 0.98
No (reference)	18 (36.00)						
**Age (years)**							
20–30	10 (41.67)	1.41	0.68	4.29	0.038	4.11	1.08, 15.63
31–40	13 (26.53)	0.73	0.63	1.34	0.247	2.08	0.60, 7.15
≥41 (reference)	4 (14.81)						
**Sex**							
Female	19 (24.68)	−0.49	0.51	0.91	0.341	0.614	0.22, 1.67
Male (reference)	8 (34.78)						
**Education**							
<College	2 (16.67)	−0.87	0.86	1.04	0.309	0.42	0.08, 2.24
College	14 (25.93)	−0.31	0.48	0.42	0.516	0.73	0.29, 1.88
>College (reference)	11 (32.35)						
**Professional title**							
Primary	9 (52.94)	1.822	0.76	2.60	0.107	3.38	0.80, 14.81
Medium	14 (20.90)	−0.23	0.65	0.13	0.721	0.79	0.22, 2.84
Senior (reference)	4 (25.00)						
**Staff type**							
Doctor	14 (33.33)	−0.69	0.71	0.95	0.330	0.50	0.12, 2.02
Nurse	8 (16.67)	−1.61	0.74	4.71	0.030	0.20	0.05, 0.86
Medical technician (reference)	5 (50.00)						
**BMI (kg/m^2^)**							
<18.5 (thin)	1 (14.29)	0.51	1.51	0.12	0.734	1.67	0.09, 31.87
18.5 to 24 (normal)	19 (29.23)	1.42	1.08	1.71	0.191	4.13	0.49, 34.55
24 to 28 (obese)	6 (35.29)	1.70	1.17	2.12	0.145	5.46	0.56, 53.52
≥28 (obesity) (reference)	1 (9.09)						
**Smoking**							
Light	3 (60.00)	1.49	0.94	2.50	0.114	4.44	0.70, 28.17
Never (reference)	24 (25.26)						
**Alcohol drinking**							
Never	19 (26.03)	−0.42	0.52	0.66	0.417	0.66	0.24, 1.80
Former	0 (0.00)	−20.57	296.48	0.00	0.999	0.00	0.00, -
Light (reference)	8 (34.78)						
**Steps**							
<5000 steps	0 (0.00)	−20.70	13,397.66	0.00	0.999	0.00	0.00, -
5000 to 10,000 steps	10 (21.74)	−0.78	0.47	2.75	0.097	0.46	0.18, 1.15
≥10,000 steps (reference)	17 (37.78)						
**Sleeping status**							
<6 h/day	0 (0.00)	−19.70	28,420.72	0.00	0.999	0.00	0.00, -
6 to 8 h/day	23 (30.26)	0.70	0.61	1.22	0.270	1.95	0.60, 6.41
≥8 h/day (reference)	4 (18.18)						
**Stress**							
Fewer	3 (50.00)	1.18	0.91	1.67	0.196	3.25	0.55, 19.38
Moderate	16 (26.67)	0.17	0.50	0.11	0.738	1.18	0.45, 3.14
More (reference)	8 (23.53)						
**Chronic disease history**							
No	26 (28.26)	−1.01	1.09	0.86	0.354	0.36	0.04, 3.09
Yes (reference)	1 (12.50)						

Note: The odds ratio (“OR”) (unadjusted) and the 95% CI were estimated using single-factor logistic regression analysis with SPSS software.

**Table 3:** Vaccination history [n (%)].

Group	Vaccination History Control (*n* = 50) Vaccination (*n* = 50)		
	Never	1 Year	2 Years
Control	20 (40.00)	18 (36.00)	12 (24.00)
Vaccination	12 (24.00)	14 (28.00)	24 (48.00)

Note: The differences were analyzed using the chi-square test.

**Table 4:** Vaccination recommendations [n (%)].

Group	For Patients			*χ* ^2^	*p*	For Family			*χ* ^2^	*p*
	Strongly	With Reservation	No	5.47	0.06	Strongly	With Reservation	No	11.76	0.003
Control	28 (56.00)	20 (40.00)	2 (4.00)			24 (48.00)	24 (48.00)	2 (4.00)		
Vaccination	39 (78.00)	10 (20.00)	1 (2.00)			40 (80.00)	10 (20)	0 (0.00)		

**Table 5:** Muti-factor logistic regression analysis on incidence of influenza-like illness in healthcare workers.

	B	SE	Wald	p	OR	95% CI	
Vaccination (Yes)	−0.93	0.48	3.76	0.05	0.39	0.15	1.00
Staff type (Nurse)	0.09	0.36	0.07	0.78	1.10	0.54	2.23

## Data Availability

The original contributions presented in this study are included in the article. Further inquiries can be directed to the corresponding author.
